# Case-control study of risk factors for incident syphilis infection among men who have sex with men in Tokyo, Japan

**DOI:** 10.5365/wpsar.2019.10.1.003

**Published:** 2019-12-09

**Authors:** Masahiro Ishikane, Yuzo Arima, Ichiro Itoda, Takuya Yamagishi, Takuri Takahashi, Tamano Matsui, Tomimasa Sunagawa, Makoto Ohnishi, Kazunori Oishi

**Affiliations:** a Field Epidemiology Training Program, National Institute of Infectious Diseases, Tokyo, Japan.; b Division of Global Infectious Diseases, Department of Infection and Epidemiology, Graduate School of Medicine, Tohoku University, Miyagi, Japan.; c Department of Disease Control and Prevention Center, National Center for Global Health and Medicine, Tokyo, Japan.; d Infectious Disease Surveillance Center, National Institute of Infectious Diseases, Tokyo, Japan.; e Shirakaba Clinic, Tokyo, Japan.; f Department of Bacteriology I, National Institute of Infectious Diseases, Tokyo, Japan.

## Abstract

**Introduction:**

In Japan, syphilis notifications have increased. Men who have sex with men (MSM) in Tokyo have contributed substantially to the increase in syphilis notifications. We thus aimed to determine the correlates of incident syphilis among them.

**Methods:**

MSM who attended a Tokyo clinic that serves sexual minorities were recruited in a case-control study in 2015. A case was seropositive for primary/secondary/asymptomatic syphilis at enrolment visit and seronegative at prior visit or had oral ulcers positive for *Treponema pallidum* DNA at enrolment. For each case, two controls seronegative at enrolment and prior visit were selected. Using logistic regression, odds ratios (ORs) and 95% confidence intervals (CIs) were estimated to assess for correlates of case status.

**Results:**

Among 35 cases, the median age was 37 (range = 21–63) years and was similar to the 71 controls. Among HIV-positive participants (26 cases and 67 controls), cases were independently associated with higher frequency of anal or oral sex (OR = 3.4; 95% CI = 1.4–8.6; increase per category from < 1/month, ≥ 1/month but < 1/week, to ≥ 1/week) and no or inconsistent condom use during anal or oral sex (OR = 3.0; 95% CI = 1.1–8.3; increase per category from using every time, occasionally, to never), adjusted for residency and time between visits.

**Discussion:**

Modifiable behaviours were associated with incident syphilis, and dissemination of prevention messages are needed.

Syphilis, a nationally notifiable infectious disease in Japan, has seen a considerable rise in the number of notifications, (*1*, *2*) similar to many other countries globally, (*3*, *4*) including within the Western Pacific Region. (*5*-*7*) In Japan, while more recent increases in syphilis have been among heterosexual men and women, (*8*) during 2013–2014, this increase was predominantly associated with men who have sex with men (MSM). (*1*, *2*) The number of notifications among MSM remained high through 2018. Notably, both the absolute number of reported MSM and heterosexual syphilis cases and the notification rate per population have been highest in Tokyo (4 cases per 100 000 persons-years during 2012–2016), contributing one third of the national cases. (*8*, *9*)

Recent studies among MSM have reported that factors associated with syphilis were low educational attainment, sex with casual partners without a condom and coinfection with other sexually transmitted infections (STIs). (*10*-*13*) Using mobile phone applications and the Internet to seek partners has also been reported as a potential risk factor for STIs, including syphilis, among MSM. (*14*-*16*) While these and other practices have been suggested as possible contributors to the recent rise of syphilis notifications in Japan, very few studies have evaluated potential predictors for syphilis acquisition in Japan. Recently, there was a cohort study analysing the risk factors of incident syphilis infection among HIV-positive MSM in Japan, (*17*) but behavioural factors were not assessed. To better understand the syphilis outbreak among MSM in Tokyo, we conducted a clinic-based case-control study based on a self-administered questionnaire to assess the potential risk factors, including modifiable behavioural factors, for incident syphilis.

## Materials and Methods

### Study design and setting

We conducted a case-control study at Shirakaba Clinic, a clinic that serves sexual minority populations. The clinic is located in the urban area of Tokyo, and most of the patients are MSM from Tokyo and its neighbouring areas. Annually, the clinic serves about 500 HIV-positive patients, and patients with HIV-infection are advised to visit the clinic for antiretroviral therapy treatment and checkup about every three months. Other patients may make a visit if they have signs/symptoms or have concerns regarding STI acquisition.

### Sampling and study population

#### Sampling

Persons who visited Shirakaba Clinic from 1 January to 31 November 2015 and received a syphilis test based on the clinician’s evaluation were recruited. Eligible subjects were Japanese males who self-reported as MSM, aged ≥ 18 years and with sexual activity (anal and/or oral sex) with another male in the six months before study entry. Based on the recent findings from Champenois et al., (*11*) assuming 62% exposure in the controls (e.g. for unprotected sex), we estimated that a total sample size of 105 participants with a case-to-control ratio of one to two would be able to detect an odds ratio (OR) of 4.8 with 80% power. (*11*)

#### Case subjects

A case was defined as an eligible subject with evidence of recent syphilis infection. For syphilis infection, one of the following conditions based on clinical examination and laboratory evaluation was required: (1) seropositivity by nonspecific (i.e. rapid plasma reagin [RPR]) and specific (i.e. *Treponema pallidum* latex agglutination [TPLA]) treponemal tests for primary, secondary or asymptomatic syphilis (*18*, *19*) at a clinic visit at the time of study entry and seronegative based on a nonspecific treponemal test at prior visit; (2) a syphilitic lesion testing positive for *T. pallidum* DNA (*polA*/*TpN47* genes) by polymerase chain reaction (PCR) (*20*, *21*) at the enrolment visit (PCR method available on a limited basis for research). We excluded late stage and neurosyphilis cases because they likely would not reflect recent infection, and sexual activity within the recent six months would not be causally related to such infections.

#### Control subjects

A control was defined as an eligible subject without evidence of recent syphilis infection. Control subjects were seronegative by nonspecific treponemal test for syphilis at both the most recent clinic visit (i.e. time of study entry) and prior visit. For each case, two controls who visited the clinic at the time closest to the time of case detection (within a month) were selected; this was done to recruit controls in a systematic manner and to ensure the same distribution of recruitment time as cases.

### Data collection

The following data were collected from the self-administered questionnaire: (1) socio-demographic characteristics, (2) health conditions including past STI history, and (3) sexual activities in the past six months. Pre-exposure prophylaxis for HIV was not available in Japan at the time and thus not included. The questionnaire was developed based on those from recent studies (*10*, *11*) and adapted to the Japanese context. Before conducting the study, we performed a pilot study (*22*) to pretest and improve the questionnaire tool. The questionnaire was completed by the respondent in a private location to reduce social desirability bias. In addition, the following data were collected from the patients’ medical charts: (1) clinical data including the stage of syphilis (primary, secondary or asymptomatic); (2) syphilis serology; and (3) HIV-related data. Participants received an STI prevention packet as compensation.

### Laboratory testing

Shirakaba Clinic tested serum for syphilis by using the RPR test and TPLA test. The diagnosis of seropositive syphilis was based on both serum RPR value and positive TPLA result. (*23*) For those highly suspected to be primary stage syphilis based on clinical diagnosis, the clinic sent samples of the syphilitic lesions from those patients to the National Institute of Infectious Diseases (NIID) in Japan (*1*) where PCR was performed. (*20*, *21*) Patients were notified of the results in seven days, and they received empirical treatment for syphilis while waiting for the results. Patients were notified of their test results in person or by phone and offered appropriate treatment and follow-up with post-test counselling. The clinic also tested subjects’ serum for HIV by using a fourth-generation combined enzyme immunoassay and antigen screening tests.

### Statistical analysis

Descriptive analyses were conducted to assess the distribution of characteristics among cases and controls. To compare distributions, the Student’s *t*-test or the Mann–Whitney U test was used for continuous variables and the χ^2^ test or Fisher’s exact test for categorical variables. Using univariate logistic regression with odds ratios and the associated 95% confidence intervals, we assessed the association between syphilis infection and sociodemographic characteristics, health conditions including past STI history and recent sexual activities. Potential risk factors for incident syphilis hypothesized a priori based on a conceptual model (e.g. condom use, frequency of sex) were considered for inclusion in a multivariable model for risk factors adjusted for duration between entry visit and prior visit. Variables with a notably large or small OR in the univariate analysis were also considered in further exploratory analyses. Statistical significance was defined as two-sided *P*-values < 0.05. All statistical analyses were performed with the Statistical Package for Social Sciences Ver.18.0 (SPSS Inc., Chicago, Illinois, USA).

### Ethics

This study was approved by the ethics committee of the NIID in Japan (approval no: NIID-564) and was implemented in accordance with the provisions of the Declaration of Helsinki. (*24*) Informed consent was obtained from all participants in the study.

## Results

### Description of syphilis cases and controls

**Figure 1:**
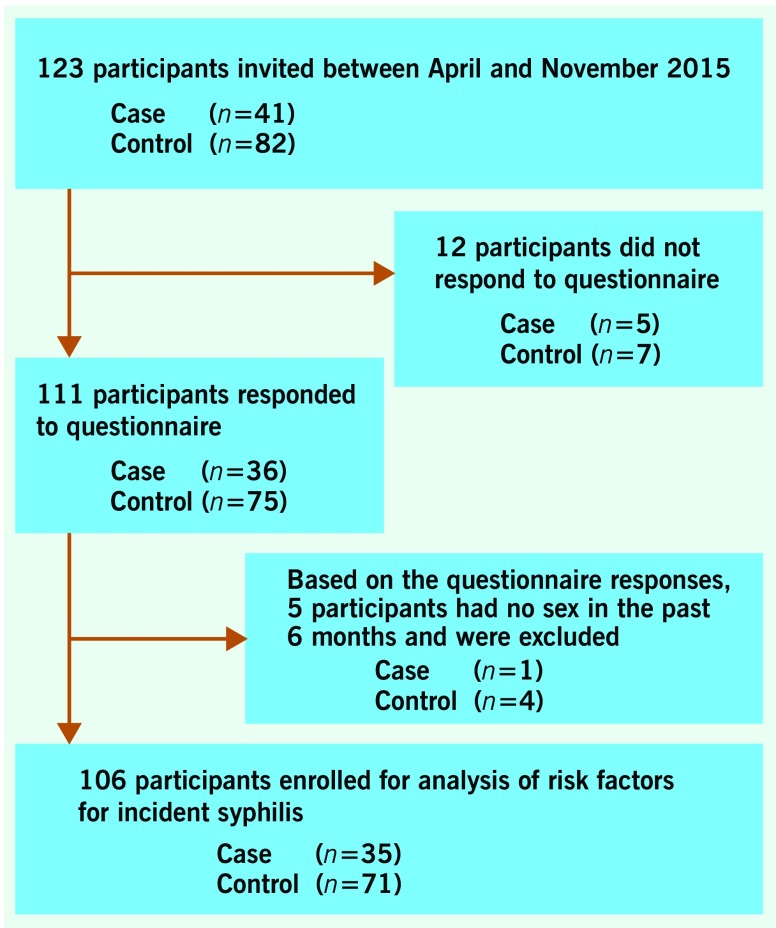
**Participant enrolment process**

During the study period, a total of 123 participants (41 cases and 82 controls) agreed to participate and were enrolled in the study. Twelve subjects agreed to participate but did not respond to the questionnaire; based on the questionnaire responses, five additional subjects were excluded because they provided discrepant answers from the recruitment screening (i.e. they denied having sex in the past six months in the questionnaire). The remaining 106 participants (35 cases and 71 controls) were enrolled for analysis (**Fig. 1**). The sexual identity of all participants was confirmed to be MSM based on self-reporting. One case was married to a woman, and one control was divorced.

[**insert** Figure 1]

Among cases, the median age at study entry was 37 (range: 21–63) years, and the median age for first sex with a male was 18 (range: 10 −25) years, both of which were similar to those of controls (**Table 1**). For stage of syphilis, three (8.6%) were primary, 13 (37.1%) were secondary and 19 (54.3%) were asymptomatic. Among the symptomatic primary/secondary cases, 12 (34.3%) presented with rash, three (8.6%) with chancre and one (2.9%) with oral ulcer. Thirty-two (91.4%) cases, including all asymptomatic cases, were diagnosed by serology, while all three (8.6%) cases with primary syphilis were diagnosed by PCR. The median duration between study entry visit and prior visit was 3.7 (IQR: 2.5–6.1) months among cases compared to 2.6 (IQR: 2.0–3.0) months among controls (*P* < 0.01).

**Table 1:** Socio-demographic and sexually transmitted infection status characteristics of study participants and association with incident syphilis by univariate analysis

-		Cases (*n* = 35), *n*(%)*		Controls (*n* = 71), *n*(%)		OR	(95% CI)	*P*-value
Socio-demographic characteristics								
Median age, years (range)		37 (21–63)		37 (23–57)		-	-	0.79
Duration between entry visit and prior visit, median month (IQR)		3.7 (2.5–6.1)		2.6 (2.0–3.0)		-	-	< 0.01
Residence		-						
-	Outside Tokyo	4	(11.4)	22	(31.0)	Ref	-	-
-	Tokyo	31	(88.6)	49	(69.0)	3.5	(1.1–11.1)	0.03
Education background		-						
-	College or university	26	(74.3)	55	(77.5)	Ref	-	-
-	High school and below	9	(25.7)	16	(22.5)	1.2	(0.5–3.0)	0.72
Employment status		-						
-	Full time	30	(85.7)	63	(88.7)	Ref		-
-	Non-full time	5	(14.3)	8	(11.3)	1.3	(0.4–4.4)	0.66
Median age at the first sex with male (range)		18 (10–25)		19 (9–30)		-	-	0.11
History of sex with female		16	(45.7)	21	(29.6)	2.0	(0.9–4.6)	0.10
Median age at the first sex with female (range)		19 (15–25)		20 (15–30)		-	-	0.32
**STI status**								
Past syphilis		24	(68.6)	32	(45.1)	2.7	(1.1–6.2)	0.02
HIV seropositive		26	(74.3)	67	(94.4)	0.2	(0.05–0.6)	0.01
Past STIs other than syphilis or HIV		31	(88.6)	42	(59.2)	5.4	(1.7–16.8)	< 0.01
-	Gonorrhoea	9	(25.7)	7	(9.9)	3.2	(1.1–9.4)	0.03
-	Chlamydia	5	(14.3)	15	(21.1)	0.6	(0.2–1.9)	0.40
-	Genital herpes	3	(8.6)	4	(5.6)	1.6	(0.3–7.4)	0.68
-	Hepatitis B	13	(37.1)	18	(25.4)	1.7	(0.7–4.2)	0.21
-	Anogential HPV	15	(42.9)	16	(22.5)	2.6	(1.1–6.2)	0.03
-	Amebiasis	3	(8.6)	3	(4.2)	2.1	(0.4–11.1)	0.39

*n **(%) unless indicated as otherwise for age, duration between study visits**

STI, sexually transmitted infection; OR, odds ratio; CI, confidence interval; HIV, human immunodeficiency virus; ART, antiretroviral therapy; IQR, interquartile range; HPV, human papillomavirus.

### Correlates of incident syphilis infection, univariate analysis

Compared to controls, cases were more likely to have resided in Tokyo (OR = 3.5; 95% CI = 1.1–11.1) and have a past history of syphilis (OR = 2.7; 95% CI = 1.1–6.2) (**Table 1**). Cases were also more likely to have a past history of STIs other than syphilis or HIV (OR = 5.4; 95% CI = 1.7–16.8); specifically, cases had a higher odds of having a past history of gonorrhea infection and anogenital human papillomavirus (HPV) infection (**Table 1**).

In contrast, while most cases and controls were infected with HIV, a lower proportion of the cases were infected with HIV relative to controls (26 cases [74.3%] versus 67 controls [94.4%]; OR = 0.2; 95% CI = 0.1–0.6) (**Table 1**). Among the HIV-positive participants, 24 cases (92.3%) and 64 controls (95.6%) were receiving antiretroviral therapy (ART) at the time of study entry and HIV-RNA was well suppressed in both cases and controls, with a median CD4 count (cell/µL) of 603 (IQR: 195–1206) in cases and 655 (IQR: 482–778) in controls. HIV-positive MSM had a shorter interval period between study entry and prior visit (median: 2.8 months; IQR: 2.1–3.5) compared to HIV-negative MSM (median: 4.9 months; IQR: 2.2–10.7). Intervals were similar for HIV-positive (median: 2.7 months) and HIV-negative (median: 2.6 months) controls, but only four out of 13 HIV-negative MSM were controls. While the majority of the HIV-positive cases were asymptomatic (18/26 cases), among the nine HIV-negative cases, six were secondary, two were primary, and one was asymptomatic for stages of syphilis.

Cases were significantly associated with both the number of sex partners and average frequency of sex (anal or oral) in the past six months compared to controls with a dose–response relationship of increased odds of case status with increase in either of these factors (**Table 2**). Cases were also associated with alcohol intake and sex toy use during sex (anal or oral). In addition, cases had a greater odds of no or inconsistent condom use during sex. While there was a greater magnitude of association for anal than oral sex, there was a dose–response trend for both; combined as a single variable (i.e. complete, occasional or no condom use based on either sex act), there was a more than 10-fold increase in odds for those that did not use condoms at all relative to those who used them every time for either sex act (**Table 2**). Although the difference was not statistically significant, cases had a fourfold higher odds of having sex with a casual partner compared to controls; all nine participants who reported a steady partner were HIV-positive, and eight of them were controls. There were no differences between cases and controls in the method of seeking partners (cruising spot, Internet or mobile phone applications) (**Table 2**). In addition to type of method, no differences were detected when comparing the number of methods used in seeking sex partners. Although there were no differences between cases and controls in the method of seeking partners, all four HIV-negative controls used mobile phone applications compared to 67% (45/67) of the HIV-positive controls.

**Table 2:** Participant characteristics regarding sexual activities in the past six months before study entry and association with incident syphilis by univariate analysis

-		Cases (*n* = 35), *n*(%)		Controls (*n* = 71), *n*(%)		OR	(95% CI)
Number of sex partners							
-	1–5	11	(31.4)	40	(56.3)	Ref	-
-	6–15	16	(45.7)	22	(31.0)	2.6	(1.0–6.7)
-	≥ 16	8	(22.9)	9	(12.7)	3.2*	(1.0–10.3)
**Partner type**							
-	Steady	1	(2.9)	8	(11.3)	Ref	-
-	Casual	34	(97.1)	63	(88.7)	4.3	(0.5–36.0)
**Method of seeking sex partners^†^**							
-	Cruising spot	23	(65.7)	47	(66.2)	1.0	(0.4–2.3)
	Internet	16	(45.7)	28	(39.4)	1.3	(0.6–2.9)
	Mobile phone applications	19	(54.3)	37	(52.1)	1.1	(0.5–2.5)
**Average frequency of sex (anal or oral)**							
-	< 1/month	4	(11.4)	24	(33.8)	Ref	
	≥ 1/month but < 1/week	21	(60.0)	40	(56.3)	3.2	(1.0–10.3)
	≥ 1/week	10	(28.6)	7	(9.9)	8.6*	(2.0–35.9)
**Alcohol intake during sex (anal or oral)**							
-	No	16	(45.7)	47	(66.2)	Ref	-
	Yes	19	(54.3)	24	(33.8)	2.3	(1.0–5.3)
**Recreational drug use during sex (anal or oral)**							
-	No	30	(85.7)	61	(85.9)	Ref	-
	Yes	5	(14.3)	10	(14.1)	1.0	(0.3–3.2)
**Sex toy use during sex (anal or oral)**							
-	No	24	(68.6)	60	(85.7)	Ref	-
	Yes	11	(31.4)	10	(14.3)	2.8	(1.0–7.3)
**Condom use during anal sex^‡^**							
-	Every time	9	(25.7)	35	(50.0)	Ref	-
	Occasionally use	18	(51.4)	26	(37.1)	2.7	(1.0–6.9)
	No use	8	(22.9)	9	(12.9)	3.5	(1.0–11.5)
**Condom use during oral sex^‡^**							
-	Every time	12	(34.3)	32	(45.7)	Ref	-
	Occasionally use	8	(22.9)	20	(28.6)	1.1	(0.4–3.1)
	No use	15	(42.9)	18	(25.7)	2.2	(0.9–5.8)
**Condom use during anal or oral sex^‡^**							
-	Every time	3	(8.6)	22	(31.4)	Ref	-
	Occasionally use	27	(77.1)	45	(64.3)	4.4	(1.2–16.1)
	No use	5	(14.3)	3	(4.3)	12.2*	(1.9–79.4)

*Significant for trend.

†Includes multiple responses

‡Data for 1 control not available for analysis

OR, odds ratio; CI, confidence interval

### Identification of potential risk factors among HIV-positive cases and controls by multivariable analysis

Given that there were only 13 HIV-negative subjects (9/35 cases and 4/71 controls) and HIV-positive subjects differed in health-care access behaviour (e.g. shorter time between visits), the main analysis was restricted to HIV-positive cases (*n* = 26) and controls (*n* = 67). Results from univariate analysis for this restricted population were similar, with case status remaining strongly associated with the following:

- Tokyo residency (OR = 3.5; 95% CI = 1.0–13.0);
- past history of syphilis (OR = 4.9; 95% CI = 1.7–14.5);
- past history of STIs other than syphilis or HIV (OR = 5.5; 95% CI = 1.5–20.1);
- number of sex partners (relative to 1–5 partners, OR = 2.1 [95% CI = 1.1–3.9] for 6–15 partners, and OR = 4.2 [95%CI = 1.2–14.8] for ≥ 16 partners);
- average frequency of sex (relative to < 1/month, OR = 4.8 [95% CI = 1.0–23.0] for ≥ 1/month but < 1/week, and OR = 15.3 [95% CI = 2.6–91.9) for ≥ 1/week]; and
- no or inconsistent condom use during anal or oral sex (relative to using every time, OR = 2.9 [95% CI = 0.8–11.1] for occasional use and OR = 8.9 [95% CI = 1.3–61.1) for no use]).
- While not significant, case status also remained associated with alcohol intake (OR = 2.0; 95% CI: 0.8–5.1) and sex toy use (OR = 2.8; 95% CI = 0.9–8.4).

Number of sex partners, past syphilis infection, past STIs other than syphilis or HIV, alcohol intake and sex toy use did not remain significant, and the association with case status was reduced when adjusted for average frequency of sex (anal or oral), an a priori hypothesized strong risk factor for incident infection. In multivariable analysis, average frequency of sex (anal or oral) and no or inconsistent condom use during either anal or oral sex both independently remained significantly associated with incident syphilis, with both having a threefold increase in odds for increase per category, adjusted for Tokyo residency and duration between entry visit and prior visit (**Table 3**).

**Table 3:** Multivariable analysis of factors associated with incident syphilis among HIV-positive participants, *n* = 93

-	Cases (*n* = 26), *n*(%)*			Controls (*n* = 67), *n*(%)		OR	(95% CI)
Tokyo residency	23	(88.5)		46	(68.7)	3.1	(0.8–12.4)
Average frequency of sex (anal or oral)	-						
< 1/month	2	(7.7)		23	(34.3)	3.4**	(1.4–8.6)
≥ 1/month but < 1/week	16	(61.5)		38	(56.7)	-	-
≥ 1/week	8	(30.8)		6	(9.0)	-	-
Condom use during oral or anal sex^†^	-						
Every time	3	(11.5)		20	(30.3)	3.0**	(1.1–8.3)
Occasionally use	19	(73.1)		43	(65.2)	-	-
No use	4	(15.4)		3	(4.5)	-	-
Duration between entry visit and prior visit, median month (IQR)	3.2	(2.5–5.8)		2.7	(2.0–3.0)	1.0***	(0.9–1.1)

*n **(%) except for duration between study visits, as indicated.**

**Odds ratio represents increase per category.

***Odds ratio represents increase per month.

†Data for 1 control not available for analysis.

OR, odds ratio; CI, confidence interval; HIV, human immunodeficiency virus; IQR, interquartile range.

## Discussion

Our case-control study found that no or inconsistent condom use during anal or oral sex and higher frequency of sex were potential risk factors for incident syphilis infection among HIV-positive MSM in Tokyo with a dose–response trend. As most of the participants were HIV-positive MSM (87.8% [93/106]), and as HIV-positive patients were believed to differ from HIV-negative patients as a population, the primary analysis was restricted to the former. As HIV patients are requested to visit this clinic on a regular basis (usually every three months, including for ART and laboratory testing), we found that the interval between visits was short among HIV-positive participants, thus increasing the likelihood of categorizing them as a control due to more frequent outcome detection. Furthermore, although the majority of the HIV-positive cases were detected as asymptomatic, the majority of HIV-negative cases were detected as having secondary syphilis; given the more routine visits among HIV-positive cases, HIV-negative cases appeared to be detected at a later, symptomatic stage of syphilis. This restriction was thus deemed important to reduce sparse data bias and detection-related bias.

Although past studies from other countries have reported similar findings, (*10*-*13*) to our knowledge, this is the first study in Japan to assess for modifiable behaviours for incident syphilis infection among HIV-positive MSM. Similar to a recent case-control study in France (*11*) that found unprotected oral sex to be a potential risk factor for incident syphilis among MSM, we also found unprotected sex to be associated with greater odds of incident syphilis; importantly, we found a strong dose–response relationship and the association to be strongest when there was no condom use for either oral or anal sex. Although there were only 13 HIV-negative participants, none of the nine cases used condoms at every sexual encounter while two of the four controls used them consistently, indicating a similar direction of risk with unprotected sex. As the majority of HIV-negative participants were also detected at the secondary stage with longer duration between visits, encouraging more routine testing among this subpopulation would be important to consider.

Our study showed that Tokyo residency was marginally associated with incident syphilis. Tokyo has been the epicentre of the syphilis outbreak in Japan with both the highest absolute number of cases and the highest notification rate in Japan. (*2*) As Tokyo residency remained associated with case status adjusted for frequency of sex, we considered that one of the possible reasons might be due to the syphilis status of the partner he would have encountered in Tokyo. As the syphilis notification rate has been highest in Tokyo, the prevalence would also be expected to be high, and, all else being equal, the risk of syphilis infection might be higher when encountering a “new partner” in Tokyo. New partner acquisition has been associated with incident STI such as anogential HPV. (*25*)

This study has several important limitations. First, this study was conducted at a single clinic in Tokyo, and generalizability of findings may be limited. However, this clinic reported the largest number of syphilis cases in Tokyo, representing 18% (76/417 cases) of cases notified from Tokyo in 2013. (*26*) The clinic-based controls also accounted for biases associated with health care–seeking behaviours and helped ensure that cases and controls arose from the same population. While restriction to HIV-positive patients was deemed important to prevent detection-related biases, our interpretations are limited to HIV-positive MSM, and the small number of HIV-negative patients precluded reliable assessments for effect modification. Second, this study may be affected by residual or unmeasured confounding, such as that due to sex with a new partner. Third, we could not evaluate temporality of events given the interval-censored nature of the data collected; although we assessed sexual activity in the six months before incident infection detection, it is not possible to determine the specific timing of sexual activity that would have been causally related to the acquisition of syphilis. Lastly, because cases and controls were aware of their syphilis infection status at the time of the survey, such knowledge could have affected the reporting behaviour differently between cases and controls.

This study was the first case-control study to evaluate potential behavioural risk factors for incident syphilis infection in Japan. In addition to locations in the Region reporting a high burden of syphilis. (*5*) These findings may be of particular relevance for Member States where a high syphilis burden has been found in HIV-positive MSM. (*27*) Although previous studies have pointed out that consistent condom use may protect against syphilis infection, (*10*-*13*) this study was the first from Japan to evaluate such practices and found that consistent condom use was associated with lower odds of incident syphilis in the current outbreak. Conversely, higher frequency of sex was associated with incident syphilis. These findings are important for making informed, evidence-based recommendations regarding syphilis prevention among MSM in Japan. Feedback of the preliminary findings to stakeholders, including the MSM community, has taken place, and reporting of HIV status became required for national syphilis surveillance in January 2019; however, concerns remain as syphilis notifications among MSM remain high. To tackle the syphilis outbreak, targeted STI prevention education (including condom use), risk communication and outreach for testing and further investigations are needed among MSM in Tokyo, particularly among non-HIV-positive MSM where information is limited. Tokyo prefecture has enhanced STI education and awareness activities, and several local jurisdictions in Japan have initiated active contact-tracing investigations and partner notifications. With the upcoming Tokyo Olympic/Paralympic Games in 2020, a major global event with a potentially unprecedented number of visitors to enter Japan, continued syphilis prevention and control will be important.
